# Correlates of HIV infection among transgender women in two Chinese cities

**DOI:** 10.1186/s40249-018-0508-2

**Published:** 2018-12-01

**Authors:** Duo Shan, Mao-He Yu, Jie Yang, Ming-Hua Zhuang, Zhen Ning, Hui Liu, Lu Liu, Meng-Jie Han, Da-Peng Zhang

**Affiliations:** 1 National Center for AIDS/STD Control and Prevention, Chinese Center for Disease Control and Prevention, Beijing, 102206 People’s Republic of China; 2 Division of AIDS Control and Prevention, Tianjin Centers for Disease Control and Prevention, Tianjin, 300011 People’s Republic of China; 3 Shenlan Public Health Consultation Service Center in Tianjin, Tianjin, 300171 People’s Republic of China; 4 Division of AIDS Control and Prevention, Shanghai Municipal Center for Disease Control and Prevention, Shanghai, 200336 People’s Republic of China

**Keywords:** HIV, AIDS, Men who have sex with men, Transgender women, Substance use, Risk behaviour

## Abstract

**Background:**

In an era when HIV transmission has been on the rise among men who have sex with men (MSM), transgender women may play a considerable role in China’s current HIV epidemic as a potential “bridge” of HIV transmission between homosexual and heterosexual populations. We sought to understand the risk behaviours and factors associated with HIV infection among transgender women in two cities in China.

**Methods:**

From January to December 2016, we recruited transgender women with the help of community-based organizations (CBOs) through a wide range of methods, including snowball sampling. After recruitment, we asked participants to fill out a structured questionnaire including questions about socio-demographics, sexual behaviours, condom use, substance use and uptake of health care services. HIV infection status was determined by using two different rapid testing reagents.

**Results:**

Among 498 subjects enrolled in this study, 233 were from Shanghai and 265 were from Tianjin. The median age was 30 years (range: 18–68; IQR: 24–33). Of them, 337 (67.7%) preferred feminine dress, 13 (2.6%) had undergone transsexual operation and 68 (13.7%) had used hormones for transition purposes. Nearly half (45.6%) reported having regular partners, and 351 (70.5%) had casual partners. Regarding condom use, 81.5% reported not always using condoms with stable partners, and 70.9% reported not using condoms with casual partners. Twenty-five (5.0%) had a history of buying sex and fifty-one (10.2%) had a history of selling sex in the past three months. A total of 200 (40.2%) participants had used at least one kind of controlled substance in the past six months. The most commonly used substances were amyl nitrates (rush popper) (99.5%) and 5-MeO-DiPT (20.0%). Among rush popper users, 170 (85.4%) reported always having sex while on the drug, and 177 (88.9%) reported increased sexual pleasure after using the drug. The HIV infection risk factors identified in our study were being located in Shanghai (a*OR* = 9.35, 95% *CI* = 3.89–22.49), selling sex in the past three months (a*OR* = 3.44, 95% *CI* = 1.31–9.01), and substance use in the past six months (a*OR* = 5.71, 95% *CI* = 2.63–12.41).

**Conclusions:**

Transgender women bear a high HIV burden in the two Chinese cities. Those involved in commercial sex tended to have inconsistent condom use, leading to high risk of HIV infection. Substance use was an independent risk factor of HIV infection by increasing sexual activities and unprotected sex, which indicated an aggravated and complex situation with possible interacting syndemic factors that could cumulatively facilitate sexual risk behaviours and HIV infection in transgender women. There is an urgent need for innovative and appropriate HIV prevention programmes targeting this unique population. Efforts should be made to provide them with tailored services including persuasive communication on consistent condom use, substance use counselling and related referral services, all with the goal of reducing HIV epidemic among transgender women.

**Electronic supplementary material:**

The online version of this article (10.1186/s40249-018-0508-2) contains supplementary material, which is available to authorized users.

## Multilingual abstracts

Please see Additional file 1 for translations of the abstract into the five official working languages of the United Nations.

## Background

The transgender population is a hidden sexual minority, composed of a diverse group of individuals who feel their gender is something other than the one they were assigned at birth [1]. In this article, we use the term “transgender women” to denote those designated male at birth who identify themselves as women among men who have sex with men (MSM) whether or not they have engaged in gender transition or enhancement procedures. Available data show that self-identifying transgender women represent over 10% of the total MSM population [2]. For a long time transgender women, a sub-group with different behavioural characteristics, have been included with MSM on behavioural and disease surveillance studies but have not received targeted intervention. Since 2007, when sexual transmission overtook injection drug use in China as the dominant mode of HIV transmission (38.9% for sexual transmission vs 29.2% for injection drug use), the proportion of homosexual transmission has shown a consistent upwards trend. Of the annual new cases diagnosed between 2007 and 2016, the proportion of homosexual transmission increased markedly from 3.4% in 2007 to 27.6% in 2016 [3–5]. Given the rapidly and continually escalating HIV epidemic among MSM, the Chinese government issued the State Council’s Five-Year Plan of Action for HIV Prevention (2016–2020) in 2017. This plan emphasized more effective and targeted interventions among MSM, and clearly specified that HIV high risk behaviours among this population should be reduced more than 10% by 2020 [6].

To achieve this goal, it is necessary to analyse the HIV epidemic among MSM in-depth. In fact, the risk behaviours of transgender women, a group that is often misclassified as the general MSM, have been neglected over the years. In international research studies, HIV prevention among transgender women has attracted much attention and the extent of the studies varies in different regions. Previous studies indicated that transgender women have a higher risk of HIV infection compared with the general MSM population, in part due to gender identity issues, social and economic levels, commercial sex and other risky behaviours, and family and social discrimination [7]. A systematic review of transwomen sex workers revealed a 4-fold higher risk for HIV compared to non-transwomen sex workers [8]. Meanwhile, high rates of drug use in this population have also been reported. The reported proportion of transgender women using drugs range from 12 to 34% [9]. Data from studies of transgender populations in the United States revealed syndemic dynamics that facilitated high-risk sexual behaviours and HIV transmission [10]. Available studies demonstrated an HIV prevalence of 27.7% among this population in the US, 21.6% in Pakistan, approximately 11–13% in Thailand, 25–34% in Jakarta and 32.2% in Peru [11–14].

Yet data on the prevalence of HIV among transgender women in China are still lacking, and the few published studies among Chinese transgender women only focused on cross-dressers involving in sex work with relatively small sample sizes, which is understandable since it would be relatively easy to identify them from appearance and recruit them into studies; also, these programmes only sought to understand the sexual behaviours and characteristics of these individuals without any further information on HIV rates [15–17]. Based on existing limited surveys, this specific group of transgender women has been characterized as male but often dressed as female, most of them were involved in commercial sex with preference of servicing heterosexual men, and also MSM. Anal sex services were mostly provided in entertainment clubs, urban fringe, parks and other hidden areas. More importantly, in recent days, many experts have indicated that this population may play a considerable role in China’s current HIV infection as a potential “bridge” of HIV transmission between homosexual and heterosexual populations.

In an era when HIV transmission has been on the rise among MSM, and given the necessity and importance of more targeted interventions among this population, efforts to better understand the transgender women population are needed in order to develop better interventions. Unfortunately, to our knowledge studies that could reliably represent the population of transgender women in China have yet to be conducted or reported in peer-reviewed literature, largely because this is a hard-to-reach and often hidden population. Current understanding about transgender women may not reflect the true situation. Meanwhile, there is still no research evidence documenting HIV rates among Chinese transgender women. This data is essential to inform future HIV intervention strategies. In this study, supported by the M.A.C. AIDS Fund, we collaborated with Chinese community-based organizations (CBOs) to recruit MSM with a focus on transgender women. We anticipate that our study will inform how best to focus future HIV prevention efforts among transgender women in China.

## Methods

### Study site

This cross-sectional study was conducted in two cities (Shanghai and Tianjin) in China from January to December, 2016. Study cities were selected mainly based on the capacity of local CBOs in reaching transgender women. Several criteria were considered when selecting the city: (1) local Centre for Disease Control and Prevention (CDC) had a close working relationship with local CBOs; (2) local CBOs had demonstrated capacity and rich work experience with HIV prevention programmes for MSM; (3) local CBOs had some preliminary field work experience with transgender women, and were willing to actively cooperate with China CDC to investigate this population.

The basic situation of local CBOs in both cities and their roles in HIV prevention are described as follows:

### Shanghai commercial sex workers (CSW) & MSM center

The organization was established and registered as a non-government organization in 2004. It mainly conducts outreach, testing and training andmaintains regular communications with sex workers and MSM every week, keeping track of their problems and needs and providing relevant consultation and health services, especially one-to-one, privacy-protected and professional counselling and HIV rapid testing. It also provides a variety of rights and health trainings for sex workers and MSM on a regular basis.

### Tianjin Shenlan public health consultation service center

It is a non-profit social organization established in 2004 and was registered with local civil affairs in 2014. It focuses on Lesbians, Gays, Bisexuals, Transgender (LGBT) community culture development, policy advocacy, youth health education, HIV counselling and rapid testing, CBOs capacity building, and technical support. Both organizations have established good relationship with the transgender community, which is critically important for recruitment process of our research.

### Recruitment methods

All participants enrolled in the study met the three minimum inclusion criteria: (1) aged 18 and above; (2) had anal or oral sexual behaviour with a male partner in the past six months; and (3) were willing to provide informed consent and take HIV tests. Local CBOs played a key role in this process by offering HIV interventions and testing mobilization through peer education, outreach services, and web-based interventions.

Specifically, we recruited transgender women in cooperation with CBOs through a wide range of methods. First, snowball sampling method was used to recruit the study subjects through the following process: CBO staff contacted key informants of the transgender women community and asked them to mobilize their peers to join our study. In order to encourage the participants to recommend and mobilize their peers to participate in our programme, we made simplified information, education and communication (IEC) brochures on HIV prevention and appointment cards, and then disseminated to them to facilitate interpretation of the programme for their peers. The mobilized peers, after completing the on-site investigation process, continued to mobilize their peers, and the cycle was thus repeated. Throughout the course of this study, we emphasized the importance of appointment mechanism based on the actual situation on each study site and the number of appointments depended on the workload of the sites. Second, outreach methods were used to recruit cross-dressers who performed as actresses in gay entertainment venues and to recruit transgender women sex workers in venues where they tended to gather, such as urban fringe and construction sites. In addition, social media were extensively applied by sending messages on HIV testing in network groups, chatting with visitors of MSM chat rooms and specifically with potential transgender women on applications including QQ (Version 8.9, Tencent, Shenzhen, China), WeChat (Version 6.7.3, Tencent, Shenzhen, China), and Blued (Version 6.6.3, Blue city, Beijing, China).

To minimize possible selection bias, once a potential participant was approached, he will be asked if his psychological gender is different from his physical gender. If the self-identified sexual identity was uncertain, they would be determined by their preference in feminine dress and by asking their experience of breast augment surgery, reproductive system reconstruction and other gender-related surgery, and hormone use, etc. If they met one of the above classifiable criteria, they would be included as transgender women. Participants who did not meet selection criteria were referred to local Voluntary Counselling and Testing (VCT) clinics.

Throughout the process, CBOs were responsible for advocacy, recruitment and on-site investigations, and CDCs were responsible for on-site quality control. Before the investigation, the CBOs were given strict and focused training on survey content, methods, and skills. After the investigation, local CDCs conducted the on-site check of the questionnaires, and corrected them in a timely manner once problems were found. In addition, staff from China CDC randomly selected and re-examined the questionnaires to check the data for consistency and accuracy.

### Data collection

Recruited participants were informed of the nature and purpose of the study and survey procedures, and asked to sign an informed consent form. Participants were also told of the risks and benefits of the programme (including referrals for other services needed), and their ability to cease participation at any time without penalty. Each participant was asked to fill out a structured questionnaire which consisted of two parts. The first part collected information on socio-demographic background, sexual behaviours, condom use, substance use, as well as STI history. This part was completed either by the participant or by the CBOs’ staff while they interviewed the participant, which took between 30 and 40 min to complete. The second part included the results of HIV and syphilis screening and confirmatory testing. Each questionnaire was given a unique identification number. For quality control purpose, standardized training was carried out for all CBO staff who were responsible for recruiting and interviewing the participants. All participants were provided with HIV post-test counselling and HIV-positive participants were referred to their local CDCs for free counselling and evaluation for treatment.

### HIV testing services

#### HIV testing

In our study, CBOs provided HIV rapid tests for MSM with technical support from the local CDCs. The rapid tests were conducted using two kinds of reagents: finger prick test kits (the Determine HIV-1/2® Rapid Test, Abbott Laboratories, Chicago, USA) and oral test kits (the Aware Determine HIV-1/2® Rapid Test, Calypte Biomedical Corporation, Pleasanton, USA). If the result of rapid tests were negative, it was deemed as negative; and if one of the results was reactive, it was then checked by another kind of rapid test kit; when both of the results were positive, it was deemed as HIV positive.

#### Data analysis

Data was double entered and crosschecked with EpiData 3.1 (EpiData Association, Odense, Denmark) to establish the database and statistical analysis was performed using SPSS 19.0 (SPSS Inc., Illinois, USA). Frequency distribution and percentages were calculated for categorical variables; median and interquartile range (IQR) were calculated for continuous variables with non-normal distribution. Pearson’s chi-square test was used to compare the socio-demographic characteristics including age group, marital status, residency, education, occupation, and average monthly income in two study sites. In addition, feminine dress preference, gender-related surgery, and hormone use were also compared to see the distribution consistency of this population across the two study sites. The chi-square test was employed in the univariable analysis to examine the factors associated with HIV infection by computing crude odds ratios (c*OR*s) with 95% confidence intervals (*CI*s). We further analysed independent risk factors of HIV infection among transgender women using Multivariable logistic regression analysis through stepwise elimination to adjust for confounders, and adjusted *OR*s (a*OR*s) with 95% *CI*s were calculated. Variables were chosen to enter into the multivariate model based on the result of univariable analysis, and together with those from a priori hypothesis from literature. These included study sites, age group, local residency, education, buying sex in the past three months, selling sex in the past three months, substance use in the past six months, and ever had discrimination-related experience in hospitals. Missing values were treated as separate categories for clarity, but otherwise were not treated differently. All testing was two-sided with statistical significance defined at the level of 0.05.

## Results

### Socio-demographic characteristics

Among the 498 participants enrolled in this study, 233 (46.8%) were from Shanghai and 265 (53.2%) were from Tianjin. The median age was 30 years (range: 18–68 years; IQR: 24–33). Most of the subjects (395, 79.3%) were single, with over 13 years’ education (284, 57.0%), and held a full-time job (305, 61.2%). There were 62.4% of participants who reported a monthly income between 3001 and 8000 Yuan (equivalent to USD 477–1270). Most of them (67.7%) preferred feminine dress, and the proportion of gender-related surgery and hormones use were 2.6 and 13.7%, respectively. Of note, 112 (22.5%) desired to have breast augmentation surgery and 160 (32.1%) desired to have gender reassignment surgery. Significant differences were observed between participants from two cities in “marital status (*χ*^2^ = 13.53, *P* < 0.05)”, “local residency” (*χ*^2^ = 45.7, *P* < 0.05) and “average monthly income” (*χ*^2^ = 36.4, *P* < 0.05). A relatively higher proportion of “cohabited” was seen in Shanghai; while there was a higher proportion of “local residents” and “participants with an average monthly income ≤ RMB 3000” in Tianjin than that in Shanghai. Detailed data are presented in Table 1.

**Table 1:** Socio-demographic characteristics (*n* = 498)

Variables	Shanghai (*n,* %)	Tianjin (*n*, %)	Total (*n*, %)	*χ* ^2^	*P*
Age group					
≤ 30	141 (60.5)	184 (69.4)	325 (65.3)	4.36	0.11
31–40	64 (27.5)	57 (21.5)	121 (24.3)		
≥ 41	28 (12.0)	24 (9.1)	52 (10.4)		
Marital status					
Single	177 (76.0)	218 (82.3)	395 (79.3)	13.53	< 0.05
Married	27 (11.6)	29 (10.9)	56 (11.2)		
Cohabited	16 (6.9)	2 (0.8)	18 (3.6)		
Divorced	13 (5.6)	16 (6.0)	29 (5.8)		
Local residency					
Yes	104 (44.6)	197 (74.3)	301 (60.4)	45.76	< 0.05
No	129 (55.4)	68 (25.7)	197 (39.6)		
Education					
≤ 9 years	39 (16.7)	30 (11.3)	69 (13.9)	4.28	0.12
10–12 years	71 (30.5)	74 (27.9)	145 (29.1)		
> 13 years	123 (52.8)	161 (60.8)	284 (57.0)		
Occupation					
Unemployed	34 (14.6)	26 (9.8)	60 (12.0)	4.05	0.26
Students	14 (6.0)	18 (6.8)	32 (6.4)		
Full-time job	144 (61.8)	161 (60.8)	305 (61.2)		
Part-time job	41 (17.6)	60 (22.6)	101 (20.3)		
Average monthly income (RMB)					
≤ 3000	45 (19.3)	86 (32.5)	131 (26.3)	36.41	< 0.05
3001–8000	142 (60.9)	169 (63.8)	311 (62.4)		
> 8000	46 (19.7)	10 (3.8)	56 (11.2)		
Feminine dress					
Yes	152 (65.2)	185 (69.8)	337 (67.7)	1.19	0.28
No	81 (34.8)	80 (30.2)	161 (32.3)		
Gender-related surgery (not including breast augmentation surgery)					
Yes	6 (2.6)	7 (2.6)	13 (2.6)	0.001	0.97
No	226 (97.4)	258 (97.4)	484 (97.4)		
Hormone use					
Yes	36 (15.5)	32 (12.1)	68 (13.7)	1.2	0.27
No	197 (84.5)	233 (87.9)	430 (86.3)		

### HIV-related sexual risk behaviours among transgender women

The median age of sexual debut among the participants was 20 years (range: 12–40 years; IQR: 17–22). Among 498 transgender women, 32 (6.4%) had participated in group sex in the past 3 months, while 227 (45.6%) had stable partners and 351 (70.5%) had casual partners in the past three months. Regarding condom use, 81.5% reported not always using condoms with stable partners, and 70.9% reported not using condoms with casual partners. Twenty-five (5.0%) had a history of buying sex in the past three months and 20 of them did not insist on using condoms. Fifty-one (10.2%) had a history of selling sex in the past three months and 43 of them did not insist on using condoms. Over a half (51.0%) had an average price for selling sex between RMB 101–500. In addition, 43 (84.3%) sold sex with heterosexual men by dressing as female. See Table 2.

**Table 2:** Risky sexual behaviors among study participants (*n* = 498)

Variables	No. of participants	%
Group sex in the past 3 months		
Yes	32	6.4
No	466	93.6
Had stable partner(s) in the past 3 months		
Yes	227	45.6
No	271	54.4
Frequency of condom use in anal sex with stable partner in the past 3 months		
Never	140	61.7
Occasionally	19	8.4
Often	26	11.4
Always	42	18.5
Had casual partner(s) in the past 3 months		
Yes	351	70.5
No	147	29.5
Frequency of condom use with casual partners in the past 3 months^a^		
Never	4	1.2
Occasionally	68	20.8
Often	160	48.9
Always	95	29.1
Buying sex in the past 3 months		
Yes	25	5.0
No	473	95.0
Frequency of condom use when buying sex in the past 3 months^a^		
Never or Occasionally	3	12.5
Often	17	70.8
Always	4	16.7
Selling sex in the past 3 months		
Yes	51	10.2
No	447	89.8
Average price for selling sex^a^		
≤ 100	19	38.8
101–500	25	51.0
> 500	5	10.2
Frequency of condom use when selling sex in the past 3 months^a^		
Occasionally	7	14.0
Often	36	72.0
Always	7	14.0
Selling sex to heterosexual men by dressing as female		
Yes	43	84.3
No	8	15.7

^a^There are missing values for the variables

### Substance use among transgender women

A total of 200 (40.2%) of the transgender women had used at least one kind of psychotropic substance, out of whom 199 used amyl nitrates, or rush poppers, in the past six months. The most commonly used substances were rush popper (99.5%) and 5-MeO-DiPT (20.0%). Among the popper users, 170 (85.4%) reported always having sex while on the drug and none reported no sexual behaviour when using it. The proportions of experiencing increased sexual pleasure and sexual desire after using the drug were 88.9 and 84.9%, respectively. And there were 68.3% of popper users reported to have an extended sexual duration after using them. Ninety-two (46.2%) reported always using condoms on poppers while 72 (36.2%) reported reduced condom use after using poppers. See Table 3.

**Table 3:** Substance use among study participants (*n* = 498)

Variables	No. of participants	%
Ever used at least one kind of psychotropic substances in the past 6 months		
Yes	200	40.2
No	298	59.8
Types of the substances		
Rush popper	199	99.5
5-MeO-DiPT	40	20.0
Methamphetamine + Cocaine	13	6.5
Others	10	5.0
Frequency of using rush poppers in the past 6 months		
< 1 time per month	55	27.6
1–3 times per month	95	47.7
≥ 1 time per week	49	24.6
Sexual behavior whenever using rush popper		
Occasionally	7	3.5
Usually	22	11.1
Always	170	85.4
Self-reported sexual pleasure after using rush popper		
Increased	177	88.9
Reduced	4	2.0
No Change	7	3.5
Uncertain	11	5.5
Self-reported sexual desire after using rush popper		
Increased	169	84.9
Reduced	4	2.0
No Change	17	8.5
Uncertain	9	4.5
Compared with not using rush popper, self-reported sexual duration when using it		
Extended	136	68.3
Shortened	9	4.5
No Change	34	17.1
Uncertain	20	10.1
Compared with not using rush popper, self-reported condom use when using it		
Never used	3	1.5
Sometimes used with increased times	1	0.5
Sometimes used with reduced times	72	36.2
Sometimes used with same times	31	15.6
Always used	92	46.2
The most frequent circumstances of using rush popper		
Providing sexual services	4	2.0
For fun	182	91.5
Both	13	6.5

### HIV prevalence and risk factors associated with HIV infection among transgender women

The prevalence of HIV among transgender women in our study was 7.6% (38/498), with 3.4% (9/265) in Tianjin and 12.4% (29/233) in Shanghai. Seven factors had statistically significant associations with HIV infection among transgender women in univariable analysis, and these included being located in Shanghai, not local residents, with an education level no more than 9 years, buying sex in the past three months, selling sex in the past three months, substance use in the past six months, and ever had discrimination-related experience in hospitals (*P* ≤ 0.05, see Table 4). In the multivariable analysis, the HIV infection risk factors identified were being located in Shanghai (a*OR* = 9.35, 95% *CI*: 3.89–22.49), selling sex in the past three months (a*OR* = 3.44, 95% *CI*: 1.31–9.01), and substance use in the past six months (a*OR* = 5.71, 95% *CI*: 2.63–12.41). It should be noted that “Buying sex in the past three months” had relatively obvious significance in the univariable analysis but had decreased significance with *P* = 0.05 in the multivariable analysis. See Table 4.

**Table 4:** Univariable and multivariable analysis for risk factors associated with HIV infection among transgender women in two Chinese cities, 2016 (*n* = 498)

Variables	No. of participants	HIV infection rate (%)	c*OR* (95% *CI*)	*P*	a*OR* (95% *CI*)	*P*
Study sites						
Tianjin	265	3.4	1.00		1.00	
Shanghai	233	12.4	3.46 (1.49–8.01)	< 0.01	9.35 (3.89–22.49)	< 0.01
Age group						
≤ 30	325	7.1	1.00			
31–40	121	11.6	3.88 (0.51–29.40)	0.19		
≥ 41	52	1.9	6.67 (0.85–52.15)	0.07		
Local residency						
Yes	301	5.3	1.00			
No	197	11.2	2.24 (1.15–4.38)	0.02		
Education						
> 13 years	284	6.0	1.00			
10–12 years	145	6.9	1.16 (0.52–2.61)	0.71		
≤ 9 years	69	15.9	2.98(1.33–6.69)	0.01		
Buying sex in the past 3 months						
No	473	7.0	1.00		1.00	
Yes	25	20	3.33 (1.18–9.45)	0.02	3.59 (1.00–12.87)	0.05
Selling sex in the past 3 months						
No	447	6.7	1.00		1.00	
Yes	51	15.7	2.59 (1.12–5.99)	0.03	3.44 (1.31–9.01)	0.01
Substance use in the past 6 months						
Yes	298	5.0	1.00		1.00	
No	200	11.5	2.45 (1.25–4.82)	0.01	5.71 (2.63–12.41)	< 0.01
Ever had discrimination-related experience in hospitals						
No	239	3.8	1.00			
Yes	259	11.2	3.22 (1.49–6.96)	< 0.01		

## Discussion

Our study showed that HIV prevalence among transgender women in Shanghai and Tianjin were 12.4 and 3.4%, respectively; which were higher than that of general MSM in Shanghai (10.25%) and Tianjin (3.0%) [18, 19]. According to Zhou’s study, the pooled prevalence of HIV infection in general MSM in China was 6.5% [20]. In both study cities, the results were consistent with the fact that compared to the general MSM population, transgender women are at higher risk for HIV considering their risk behaviours. Studies from the US and Southeast Asia also showed disproportionately high rates of HIV among transgender persons, compared to the general MSM population [11, 21]. In our multivariable analysis, selling sex in the past three months remained significantly associated with HIV infection, as did substance use in the past six months, which were all independent factors related to HIV risk in the other studies [22, 23].

Our study demonstrated different socio-demographic characteristics among transgender women in China. Only 13.7% took hormones and even fewer (2.6%) sought the use of surgical procedures. This was not the case among transgender women in US and Thailand, where a majority of transgender people had taken hormones to enhance their gender presentation and many had engaged in surgical procedures as well [24, 25]. Although risky sexual behaviours were not defined consistently across the various studies, we found an alarmingly vast majority of transgender women who could not consistently use condoms with their stable or causal partners. Roughly 10% of transgender women engaged in commercial sex work in other studies [1, 26], in contrast to our finding of 10.2% selling sex and 5.0% buying sex. In our study, selling sex was significantly associated with an elevated risk for HIV infection, which was in accordance with other studies [27, 28]. In recent years, more and more news media in China have reported that “prostitution women” arrested by local public security departments turned out to be “male”. In fact, we found that due to the cross-dressing characteristic of this population, their recipients of sexual service usually did not know their biological gender as “male” and thought that they were “female”. As such, this population could have sexual behaviours both with heterosexual men and MSM, which made them become a population with a higher risk for HIV transmission compared to the general MSM. Based on our initial understanding of this population from the field study, they could be referred to as a group of people “whose number was not large but with extremely high risks of HIV infection and transmission”. Indeed, some studies even suggested a high awareness of HIV-related sexual risk behaviours among transgender women but a low priority of HIV prevention compared to other immediate concerns [10, 26]. Given that this was a “bridge” population of HIV transmission between homosexuality and heterosexuality and in view of the complexity of this population, future HIV routine outreach work among MSM conducted by health staff needs to pay more attention on this subset minority and focus on encouraging their negotiation of consistent condom use with all of their clients whenever involving in commercial sex.

Another unique risk behaviour of HIV among transgender women was the high rate of substance use (40.2%) in our study, which was much higher than that in two other studies among the general MSM in China [29, 30]; and even higher than transgender women population with reported rates of 30–40% in other countries [31, 32]. Our study showed that most popper users had self-reported increased sexual activity and increased unprotected sex, which suggested close interrelations between substance use, high-risk sexual behaviours and risk of HIV transmission among this population [33]. Substance use was an independent predictor of HIV infection among transgender women in our study, which indicated an aggravated and complex situation with possible interacting syndemic factors that could cumulatively facilitate sexual risk behaviours and HIV infection in this population. Studies also suggested that substance use before or during sex compromises cognitive or behavioural abilities to use condoms, and synthetic drug use tends to increase the risk of HIV transmission by reducing safe behaviour, increasing the likelihood of random sexual behaviour, and expanding the network of sexual partners [34, 35]. Future HIV prevention programmes needs to focus more on this high-risk subset of transgender women.

There was no significant association found between discrimination-related experience and HIV infection, however, over half reported having had discrimination-related experience in hospitals, which may prevent their access to available HIV services. Although there was inadequate data in our study to verify the direct relations between discrimination and HIV infection, other studies indicated that discrimination among transgender women may contribute to the worsening of their gender transition due to limited HIV prevention and low social support, and in this way, leading to the vulnerability of HIV-related risk behaviours [36, 37].

Our study has several strengths. We systematically described the socio-demographic and risk behaviour characteristics of the self-identifying transgender women, and explored the associated risk factors of HIV infection, which could provide an opportunity for understanding the epidemiology of behaviours and HIV risks among this population. In addition, since studies on Chinese transgender women population have been scarce, especially when there hadn’t been well-defined transgender population in China, in our study, CBOs were fully mobilized to utilize multiple channels to find Chinese transgender women population with a clear definition for the first time, which filled the gap in China where this marginalized population was not well understood. The CBOs’ involvement in our study strongly guaranteed the sample size of this hard-to-reach population.

The study limitations include a lack of HIV incidence data, qualitative data and an insufficient number of HIV positive samples that prevented a better understanding of risk factors among transgender women. Study participants were selected in ways that did not limit selection biases, and our results could not be nationally representative of this subgroup of MSM. Besides, considering that our subjects were of relatively hidden populations, their emotions and self-efficacy were less represented in our data compared to the behavioural information collected, limiting the richness of the analysis. However, our study does provide correlates of HIV infection that could help facilitate future HIV prevention strategies.

Despite the limitations, our study results have shown that transgender women population actually exists in various forms and statuses in China. We found that this population is not monolithic, but rather nuanced with different risk behaviours, such as involving in commercial sex and substance use. This provides a direction for future programmes targeting this high-risk and under-served population, ensuring more focused intervention.

## Conclusions

Our study suggests the urgent need for HIV prevention strategies that are relevant and appropriate in this population. Our findings revealed relatively heightened HIV risks including commercial sex and substance use among Chinese transgender women, which is unique to this population; and a higher HIV prevalence was seen in transgender women than that of the general MSM. In China, despite the high priority for HIV prevention among transgender women, this population has neither become a part of national HIV surveillance system nor received specific HIV prevention. Actually, transgender women were often included with the general MSM in HIV programmes in China [38, 39], but without targeted HIV prevention strategy for this independent subgroup of MSM. Since over a half of the transgender women in our study ever had discrimination-related experience in hospitals, which may prevent their access to comparable health services, improved health service provided by hospitals built on current HIV testing may be an important approach. As this is a relatively hidden population, the approach needs to go beyond traditional prevention methods by training the key informants and influential social network leaders of transgender women communities, not only in identifying effective strategies for reaching them but also in persuasive communication and education in response to the unique reality of this population [40, 41]. Pilot research could be considered in China and does not need to be overarching. On the contrary, preliminary programmes and services could consider establishing transgender support groups, providing them with IEC service on commercial sex, substance use counselling and referral services, all with the goal of reducing HIV epidemic among transgender women. Future efforts should be made towards the continual study on developing methods that are acceptable and accessible for them.

## Additional file

Additional file 1: Multilingual abstracts in the five official working languages of the United Nations. (PDF 265 kb)

## Abbreviations

AIDS: Acquired Immune Deficiency Syndrome
a*OR*: Adjusted *OR*
CBO: Community-based Organization
CDC: Center for Disease Control and Prevention
*CI*: Confidence intervals
c*OR*: Crude odds ratios (s)
HIV: Human Immunodeficiency Virus
IQR: Interquartile range
MSM: Men who have sex with men
STI: Sexually transmitted infection
