# Supplementary material for: Correlates of HIV infection among transgender women in two Chinese cities
Source: Infect Dis Poverty. 2018 Dec 1;7:123. doi: 10.1186/s40249-018-0508-2 (PMC6276265; doi:10.1186/s40249-018-0508-2)
Supplement: Supplementary file 1 — Multilingual abstracts in the five official working languages of the United Nations. (PDF 265 kb) [file 40249_2018_508_MOESM1_ESM.pdf]

Translation of the abstract into the five official working languages of the United Nations

معدلات الإصابة بفيروس نقص المناعة البشري لدي النساء المتحولين جنسياً في مدينتين صينيتين .

دوو شان ,ماو هي يو , منج وا شونج ,تشن ننج هوي ليو ,لو ليو
منج جي هان و دا بن شانج

الخلفية :في وقت شهدت فيه نسبة إنتقال مرض نقص المناعةإرتفاعاًشديداًبينالرجال الممارسين الجنس مع رجال (إم إس إم) ,وبالنظرللأدلة علي الدور الكبير الذي تلعبه النساء المتحولات جنسياًفي الصينفي إنتشار وباء نقص المناعة "كجسر" محتمل في إنتقال مرض نقص المناعة بين الأفراد المثليين جنسياً و المغايرين جنسياً,وقدبذلنا الكثير من الجهدلفهمالسلوك الخطيروالعوامل المرتبطة بالإصابة بمرض نقص المناعةبين النساء المتحولاتجنسياًفي إثنين من المدن الصينية.

الطرق:لقد قمنا بتجنيدعدد من النساء المتحولات جنسياً بمساعدة المنظمات المجتمعية (سي بي أو س)بإعتماد العديد من الطرق , متضمنةطريقة كرة الثلج في أخذ العينات. بعد التجنيد , نطلب من المشاركين ملء إستبيان معد سابقاً يتضمن أسئلة حول الظروف الإجتماعيةوالديموغرافية, السلوكيات الجنسية , إستعمال الواقي , تعاطي المادة المخدرةوتلقيالخدماتالصحية. يتمتحديد حالات الإصابة بنقص المناعةبواسطةإستخدام إثنين من المحاليل الخاصةبالإختبار السريع الخاص بالكشف عن فيروس نقص المناعة.

النتائج:من بين 498حالةمسجلة بالدراسة , 233(46.8%)حالة من شانغهاي و 265(53.2%) حالة من تيان شن. متوسط العمر 30عاماً (نطاق:1 8- 68 ;33--IQR:24) فضل حوالي الثلثين , أو (67.7%) ملابس نسائية . أجري 13(2.6%)عملية التحويل الجنسي وإستخدم68(13.7%)الهرمونات بهدف التحول . خلال الثلاث أشهر الماضية ,حصل حوالي النصف (45.6%) على شركاء بصفة منتظمة , و 351 (70.5%) على شركاء بصفة مؤقتة . فيما يخص إستخدام الواقي الذكري , أبلغ 81.5% عن عدم إستعمال الواقي بصورة مستمرة مع الشريك القار، و أبلغ 70.9% عن عدم إستعمال الواقي مع الشركاء المؤقتين . كان لدي خمس وعشرون(5.0%)مشاركتاريخاً في شراء الخدماتالجنسية مقابل المال خلال الثلاثةأشهر الماضية و 20 منهملم يستخدمواواقياتذكريةخلال كل لقاء جنسي . وكان لدي واحد وخمسون(10.2%) تاريخا في بيع الجنس مقابل المال خلال الثلاثأشهر الماضية و لم يصر 43 منهم علىإستخدام الواقيات الذكرية تعاطي مجموع 200 مشارك للتجربة (40.2%) علي الأقل نوع واحد من الأدوية ذات التأثير المخدر و المقننة من قبل الحكومة خلال الستة أشهر الماضية . وأكثر هذه المواد إستخداماً هو نترات الأميل(زجاجات النشوة) (99.5%و20.0%)5MeO-Dipt(مواد ذات تأثير مخدر ومسببة للهلوسة). إستخدمت الأغلبيةالساحقة من متعاطي المادة المخدرة (199/200,99.5%)زجاجات النشوة , من بينهم 170(85.4%)أقر بممارسة الجنسدائماً وهم منتشين بالعقار , وأقر177(88.9%)بنشوة جنسية عارمةبعد تعاطي المادة المخدرة . أقر متعاطو زجاجات النشوة زيادة الرغبة الجنسية بعد إستخدامها (84.9%)وحظي68.3%منهمزيادةفترة ممارسة الجنس بعدإستخدامها. أقر إثنان و تسعون(46.2%)بإستخدام الواقي الذكري بصفةمستمرة وهم منتشين بزجاجات النشوة بينما72(36.2%)أقروابإستخدامالواقي الذكري بصورة أقل بعدتعاطي زجاجات النشوة . لقد تم التعرف علي عوامل الخطر المسببة للإصابة بمرض نقص المناعة في شانغهاي من خلال دراستنا(aOR=9.35,95%CL=3.89-22.49),بيع الخدمات الجنسية مقابل المال خلال الأشهر الثلاث الماضية(aOR=3.44,95%CL=1.31-9.01),وتعاطي المادة المخدرة خلال الستة أشهر الماضية(aOR=5.71,95%CL=2.63-12.41).

الإستنتاجات :تتحملالنساء المتحولات جنسياً عبئا كبيرا في نقل مرض نقص المناعة في المدينتين الصينيتين . يميل المشاركون المنخرطون في تجارة الجنس إلي عدم إستخدام الواقي الذكري بشكل دائم , مما يؤدي إلى خطر أكبر في الإصابة بمرض نقص المناعة . يعتبر تعاطي المادة المخدرة عامل مستقل من عوامل الخطرالمسببة للإصابة بمرض نقص المناعة ,وقد أقر مستخدمو زجاجات النشوة عن زيادة النشاط الجنسي وزيادةالجنس غير الآمن , مما يشير إلي موقف معقد ومتفاقم بالمشاركة مع عدةعوامل مساعدة تسهل تراكم سلوكيات جنسية خطيرة و أيضاً خطر الإصابة بمرض نقص المناعة في النساء المتحولات جنسياً. هناك حاجة ملحة إلي برامج مبتكرة و مناسبة للوقاية من مرض نقص المناعة موجهة إلى هذه الفئة الفريدة من السكان . ينبغي بذل المزيد من الجهود لتزويدهم بالخدمات المتضمنة إقناعهم بضرورة الإستخدام الدائم للواقي الذكري كلما انخرطوا في تجارة الجنس , وتعاطي المادة المخدرة وخدمات الإحالة المتعلقة بهما, بهدف تقليل إنتشار وباء نقص المناعة بين النساء الصينيين المتحوليين جنسياً .

Translated from English version into Arabic by Bishoy ISAAC and Malika, through

中国两城市跨性别女性人群 HIV 感染状况及其相关因素研究

引言：在中国，艾滋病病毒（HIV）经男男同性性行为人群（MSM）传播持续上升，且有证据表明，跨性别女性人群作为艾滋病在同性及异性间传播潜在的"桥梁人群"，可能在中国目前的艾滋病流行中扮演重要的角色。在此背景下，我们试图了解中国跨性别女性人群 HIV 感染风险和相关因素。

方法：在两城市，通过与 MSM 社区组织（CBO）合作，采用包括滚雪球抽样在内的多种方法招募跨性别女性者参与调查。使用结构化问卷收集包括社会人口学，性行为，安全套使用，物质滥用和医疗保健服务使用相关信息。使用两种不同的快速检测试剂判定调查对象 HIV 感染状况。

结果：本研究纳入的 498 名调查对象中，233 人（46.8%）来自上海，265 人（53.2%）来自天津。中位年龄为 30 岁（范围：18-68；四分位间距：24-33）。约 2/3，即 337 人（67.7%）喜欢女性化着装。仅 13 人（2.6%）接受过变性手术，68 人（13.7%）曾使用雌激素使自己更女性化。在过去三个月中，近一半（45.6%）有固定伙伴，351（70.5%）有临时性伴。关于安全套的使用，81.5%的调查对象自我报告与固定性伴不能坚持使用安全套，70.9%表示与临时性伴不能坚持使用安全套。在过去三个月中，25 人（5.0%）有买性行为史，其中 20 人在进行性行为时没有坚持使用安全套。51 人（10.2%）有卖性行为史，其中 43 人在进行性行为时没有坚持使用安全套。在过去六个月中，共有 200 人（40.2%）使用过至少一种精神类物质。最常用的物质是 rush popper（99.5%）和零号胶囊（20.0%）。在 rush popper 使用者中，有 170 人（85.4%）报告每次使用时均发生性行为，177 人（88.9%）报告使用药物后性快感增强。84.9%报告使用药物后性欲望增强，68.3%报告使用药物后每次性活动的时间延长。92 人（46.2%）报告使用 rush popper 后发生性行为时每次均使用安全套，而 72 人（36.2%）报告使用 rush poppers 后发生性行为时使用安全套的次数减少。本研究显示，跨性别女性人群 HIV 感染的危险因素包括居住在上海（aOR = 9.35，95%CI = 3.89-22.49），过去三个月有过卖性行为（aOR = 3.44，95%CI = 1.31-9.01）和过去六个月有过物质滥用（aOR = 5.71，95%CI = 2.63-12.41）。

结论：中国两城市跨性别女性人群 HIV 感染情况严重。该人群中，从事商业性行为者坚持使用安全套比例低，呈现较高的 HIV 感染风险。物质滥用是该人群 HIV 感染的独立危险因素，大多数 rush popper 使用者自我报告性活动增加和无保护性行为增加，这表明同时存在的多种因素相互作用可能加剧和促进跨性别女性人群的 HIV 危险性行为，并使得该人群 HIV 感染复杂化。目前迫切需要针对这一特殊人群开展创新和适宜的 HIV 预防项目，为该人群提供有关服务，包括为从事商业性行为者进行有关坚持使用安全套的说服性沟通，提供物质滥用相关的咨询和转诊服务，以在减少中国跨性别女性人群的艾滋病流行。

Translated from English version into Chinese by the authors

Corrélats de l'infection par le VIH parmi les femmes transgenre dans deux villes de Chine

Duo Shan, Mao-He Yu, Jie Yang, Ming-Hua Zhuang, Zhen Ning, Hui Liu, Lu Liu,
Meng-Jie Han et Da-Peng Zhang

Contexte : À l'heure où le VIH progresse parmi les hommes ayant des relations homosexuelles, et face aux indices qui suggèrent que les femmes transgenre pourraient jouer un rôle considérable dans l'épidémie de VIH actuelle en Chine en servant potentiellement de « passerelle » de transmission du virus entre les populations homosexuelles et hétérosexuelles, nous avons cherché à comprendre les comportements à risque et les facteurs de risque associés à l'infection par le VIH chez les femmes transgenre de deux villes chinoises.
Méthodes : Nous avons recruté des femmes transgenre par l'intermédiaire d'associations locales, en employant diverses méthodes, dont l'échantillonnage « boule de neige ». Après le recrutement, nous avons demandé aux participantes de remplir un questionnaire structuré comportant des questions sur leur statut sociodémographique, leurs comportements sexuels, l'usage du préservatif, la consommation de substances et le recours aux services de santé. Le statut d'infection par le VIH a été déterminé à l'aide de deux réactifs de test rapide différents.
Résultats : Parmi les 498 sujets enrôlés pour cette étude, 233 (46,8 %) vivaient à Shanghai et 265 (53,2 %) à Tianjin. L'âge médian était de 30 ans (extrêmes : 18 et 68 ; IQR :24-33). Les deux tiers environ, soit 337 sujets (67,7 %), préféraient les vêtements féminins. Treize (2,6 %) avaient subi une opération de changement de sexe et 68 (13,7 %) avaient pris des hormones pour la transition. Près de la moitié (45,6 %) avaient eu des partenaires réguliers au cours des deux mois écoulés et 351 (70,5 %) des partenaires de passage. En ce qui concerne l'usage du préservatif, 81,5 % des participantes ont rapporté qu'elles n'en utilisaient pas toujours avec leurs partenaires stables, et 70,9 % n'en utilisaient pas avec les partenaires de passage. Vingt-cinq sujets (5,0 %) avaient acheté des faveurs sexuelles au cours des trois derniers mois, et 20 de ceux-ci n'avaient pas utilisé un préservatif lors de tous les rapports. Vingt et un sujets (10,2 %) avaient vendu des faveurs sexuelles au cours des trois derniers mois et 43 d'entre eux n'avaient pas insisté pour utiliser un préservatif. Au total, 200 participantes (40,2 %) avaient consommé au moins une sorte de substance réglementée au cours des six derniers mois, le plus souvent des nitrates d'amyle (« rush popper ») (99,5 %) et de la 5-MeO-DiPT (20,0 %). La grande majorité des consommatrices (199/200, 99,5 %) consommaient des rush poppers ; 170 (85,4 %) d'entre celles-ci ont signalé qu'elles avaient toujours des relations sexuelles lorsqu'elles étaient droguées et 177 (88,9 %) que leur plaisir sexuel était alors augmenté. Les consommatrices de poppers ont également rapporté une augmentation du désir sexuel après la consommation (84,9 %) et 68,3 % d'entre elles avaient des rapports sexuels prolongés quand elles étaient droguées. Quatre-vingt-douze participantes (46,2 %) ont rapporté qu'elles utilisaient toujours des préservatifs après avoir consommé des poppers, et 72 (36,2 %) qu'elles les utilisaient moins quand elles étaient droguées. Les facteurs de risque d'infection par le VIH identifiés dans notre étude étaient le fait de vivre à Shanghai (*aOR* = 9,35, *IC* à 95 % de 3,89 à 22,49), la vente de faveurs sexuelles au cours des trois derniers mois (*aOR* = 3,44, *IC* à 95 % de 1,31 à 9,01) et la consommation de substances au cours des six derniers mois (*aOR* = 5,71, *IC* à 95 % de 2,63 à 12,41).
Conclusions : Le fardeau du VIH pèse lourdement sur les femmes transgenre dans ces deux villes de Chine. Les participantes impliquées dans le commerce du sexe utilisaient souvent les préservatifs de façon irrégulière, d'où un risque élevé d'infection par le VIH. La consommation de

substances était un facteur de risque d'infection par le VIH indépendant. La plupart des consommatrices de « poppers » rapportaient une activité sexuelle accrue et des rapports non protégés plus fréquents, ce qui signale une situation péjorative et complexe, avec de possibles interactions de facteurs syndémiques qui pourraient favoriser les comportements sexuels à risque et l'infection par le VIH parmi les femmes transgenre. Il est urgent d'élaborer des programmes de prévention du VIH innovants et appropriés à destination de cette population particulière. Il faut s'efforcer de leur dispenser des services, notamment une communication persuasive sur l'usage constant du préservatif dès lors qu'elles participent au commerce du sexe, un conseil sur les toxicomanies et des services de renvoi correspondants, dans le but de réduire l'épidémie de VIH parmi les femmes transgenre chinoises.

Translated from English version into French by Suzanne Assenat and Isabelle Mathis, through

TRANSLATORS
WITHOUT BORDERS

Корреляты на ВИЧ-инфекцию среди трансгендерных женщин, проживающих в двух городах Китая

До Шань, Мао-Хэ Юй, Цзе Ян, Мин-Хуа Чжуан, Чжэнь Нин, Хуэй Лю, Лу Лю,
Мэн-Цзе Хань и Да-Пэн Чжан

Общие сведения: В эпоху, когда в передаче ВИЧ-инфекции наблюдается рост среди категории мужчин, имеющих половые контакты с другими мужчинами, а также принимая во внимание сведения о том, что трансгендерные женщины могут оказывать значительное влияние на текущую эпидемию ВИЧ-инфекции в Китае, выполняя функцию потенциального «моста» в передаче ВИЧ-инфекции с гомосексуальной на гетеросексуальную группу населения, мы поставили целью понять факторы рискованного поведения, равно как и факторы, связанные с ВИЧ-инфицированием среди трансгендерных женщин в двух городах Китая.

Методы: Мы привлекли к участию в исследовании трансгендерных женщин при помощи организаций местных сообществ, задействуя различные методы, включая метод «снежного кома». Затем мы попросили участниц заполнить структурированный вопросник, включающий вопросы социально-демографического характера, вопросы о сексуальных практиках, использовании презервативов, употреблении наркотических веществ и об обращениииза медицинской помощью. Определение ВИЧ-статуса проводилось с помощью двух разных экспресс-методов тестирования.

Результаты: Среди 498 обследуемых, принявших участие в данном исследовании, 233 человека (46,8%) были из Шанхая и 265 человек (53,2%) из Тяньцзиня. Медианный возраст составил 30 лет (диапазон: 18—68 лет; МКР: 24—33 лет). Около двух третей опрошенных – 337 человек (67,7%) предпочитали носить женскую одежду. Всего 13 человек (2,6%) сделали операцию по изменению пола, тогда как 68 человек (13,7%) принимали гормональные

препараты. За последние три месяца почти половина опрошенных (45,6%) имела постоянных партнеров, а 351 человек (70,5%) имели случайных партнеров. Что касается использования презервативов, 81,5% не всегда пользовались презервативами с постоянными партнерами, тогда как 70,9% не использовали презервативы со случайными партнерами. Двадцать пять (5,0%) участниц покупали сексуальные услуги за последние три месяца, а 20 из них не пользовались презервативами при каждом половом контакте. Пятьдесят одна участница (10,2%) за последние три месяца продавали сексуальные услуги, при этом 43 из них не настаивали на использовании презерватива. За последние шесть месяцев в общей сложности 200 человек (40,2%), принявших участие в исследовании, употребляли как минимум один вид контролируемого наркотического вещества. Наиболее распространённые наркотические вещества включали амилнитрит (99,5%) и 5-метокси-диизопропилтриптамин [MeO-DiPT] (20,0%). Подавляющее большинство принявших участие в исследовании (199/200, 99,5%) употребляли амилнитрит, среди них 170 (85,4%) человек сообщили, что всегда занимались сексом под воздействием данного наркотика, а 177 (88,9%) признались, что употребление наркотика повышало удовольствие от полового контакта. Употреблявшие амилнитрит также сообщали о повышении желания заниматься сексом в результате употребления препарата (84,9%), а 68,3% из них испытывали более продолжительный половой контакт после его употребления. Девяностодва человека (46,2%) признались, что всегда пользовались презервативами при употреблении амилнитрита, тогда как 72 (36,2%) отмечали, что пользовались презервативами реже после принятия наркотика. В ходе нашего исследования были выявлены следующие факторы риска ВИЧ-инфицирования в Шанхае: (*aOR* = 9,35; 95% *CI* = 3,89–22,49) продавали сексуальные услуги за последние три месяца (*aOR* = 3,44; 95% *CI* = 1,31–9,01) и употребляли наркотические вещества за последние шесть месяцев (*aOR* = 5,71; 95% *CI* = 2,63–12,41).

Выводы: Трансгендерные женщины в двух исследуемых городах Китая подвергаются значительному риску ВИЧ-инфицирования. Участницы, оказывавшие сексуальные услуги на коммерческой основе, имели тенденцию нерегулярно пользоваться презервативами, что влекло высокий риск ВИЧ-инфицирования. Употребление наркотических веществ являлось независимым фактором риска ВИЧ-инфицирования, и большинство применявших амилнитрит сами признавались в том, что имели повышенную сексуальную активность и более частые незащищённые половые контакты, что свидетельствует о серьёзной и комплексной ситуации с потенциально взаимосвязанными систематическими факторами, способными в совокупности привести к рискованному сексуальному поведению и ВИЧ-инфицированию среди трансгендерных женщин. Существует острая потребность в передовых и эффективных программах по предотвращению ВИЧ, ориентированных на эту особую группу населения. Чтобы снизить темпы распространения ВИЧ-инфекции среди трансгендерных женщин, проживающих в Китае, необходимо донести до них информацию о важности использования презервативов во время оказания сексуальных услуг, предоставить доступ к услугам психологической помощи лицам, употребляющим наркотические вещества, а также к другим услугам информационного характера.

Translated from English version into Russian by Ira Kulinevych and Liudmila Tomanek, through

Correlaciones de la Infección VIH en las Mujeres Transgénero en Dos Ciudades de China

Duo Shan, Mao-He Yu, Jie Yang, Ming-Hua Zhuang, Zhen Ning, Hui Liu, Lu Liu,
Meng-Jie Han y Da-Peng Zhang.

Contexto: En una era donde la transmisión de VIH ha ido en aumento en los hombres que tienen relaciones sexuales con hombres (HSH), y dada la evidencia de que las mujeres transgénero tienen un rol importante en la epidemia de VIH actual en China como un "puente" potencial de transmisión de VIH entre las poblaciones homosexuales y heterosexuales, buscamos entender los factores y las conductasde riesgo asociadas con la infección de VIH entre las mujeres transgénero en dos ciudades en China.

Métodos: Reclutamos a mujeres transgénero con la ayuda de las organizaciones comunitarias (OCs) a través de una gran variedad de métodos, que incluyen el muestreo de bola de nieve. Luego del reclutamiento, les pedimos a las participantes que completen un cuestionario estructurado que incluía preguntas socio -demográficas, acerca de conductas sexuales, el uso de preservativos, el consumo de sustanciasy el entendimiento delos servicios de asistencia médica. Se determinó el estado de la infección de VIHa través del uso de dos reactivos diferentes para pruebas rápidas.

Resultados: De las 498 personas registradas en este estudio, 233 (46,8%) eran de Shanghai y 265 (53,2%) eran de Tianjin. La edad media era de 30 años (rango: 18–68; IQR: 24-–33). Alrededor de dos tercios, o 337 (67,7%) preferían la indumentaria femenina. Solo13 (2,6%) se habían sometido a una operación transexual y 68 (13,7%) habían consumido hormonas con el fin de realizar la transición. En los últimos tres meses, casi la mitad (45,6%) tuvo parejas fijas, y 351 (70.5%) tuvieron parejas temporales. Con respecto al uso de preservativos, el 81,5% manifestó que no los usaban siempre con las parejas estables, y el 70,9% manifestó que no utilizaban preservativos con las parejas temporales. Veinticinco (5,0%) participantes tenían antecedentes de haber pagado por sexo en los últimos tres meses y 20 de ellas no utilizó preservativosen todos los encuentros sexuales. Cincuenta y uno (10,2%) tenían antecedentes de haber cobrado por relaciones sexuales en los últimos tres meses y 43 de ellas no habían insistido en usar preservativos. Un total de 200 (40,2%) de las participantes del estudio habían consumido al menos un tipo de sustancia controlada en los últimos seis meses. Las sustancias consumidas más comunes fueron el nitrato de amilo (popper) (99,5%) y 5-MeO-DiPT (20,0%). La gran mayoría de las que consumen sustancias (199/200, 99,5%) inhalaban poppers, entre las cuales 170 (85.4%) manifestaron tener relaciones sexuales siempre bajo los efectos de la droga, y177 (88,9%) manifestaron un mayor placer sexual luego de haber consumido la droga. Las consumidoras de popper también expresaron tener un deseo sexual mayor luego de consumir (84,9%) y 68,3% de ellas tenían una duración sexual más larga luego delconsumo. Noventay dos (46,2%) reportaron que siempreutilizaban preservativos bajo el efecto del popper mientras que 72 (36,2%) manifestaron un uso reducido de preservativos luego de consumir popper. Los factores de riesgo de VIH identificados en nuestro estudio se

encontraron en Shanghai (*aOR* = 9,35, 95% *CI* = 3.89–22.49), cobrar por relaciones sexuales en los últimos tres meses (*aOR* = 3,44, 95% *CI* = 1,31–9,01), y consumo de sustancias en los últimos 6 meses (*aOR* = 5,71, 95% *CI* = 2,63–12,41).

Conclusiones: Las mujeres transgénero cargan con un alto índice de VIH en las dos ciudades de China. Las participantes involucradas en las relaciones sexuales comerciales tendían a usar preservativos de manera irregular, lo cual lleva a un alto riesgo de infección de VIH. El consumo de sustancias fue un factor de riesgo independiente dela infección VIH,y la mayoría de las que consumieron popper habían reportado una actividad sexual alta y sexo sin protección elevado, lo que indicaba una situación compleja y agravada con la posible interacción de factoressindémicos que podrían facilitar, de manera creciente, las conductas de riesgo sexuales y la infección de VIH en mujeres transgénero. Hay una necesidad urgente de programas innovadores y apropiados de prevención de VIH que se centren en esta población única . Deberían tomarse más medidas para proporcionarles servicios que incluyan la comunicación persuasiva sobre el uso regular del preservativo cuando seinvolucren en el sexo comercial, el asesoramiento del consumo de sustancias, y los servicios de referencia relacionados. Todo esto con el objetivo de reducir la epidemia del VIH en las mujeres transgénero de China.

Translated from English version into Spanish by Daria and Liudmila Tomanek, through
